# Supplementary material for: In Vitro and In Vivo Effect of Amikacin and Imipenem Combinations against Multidrug-Resistant E. coli
Source: Trop Med Infect Dis. 2022 Oct 2;7(10):281. doi: 10.3390/tropicalmed7100281 (PMC9609961; doi:10.3390/tropicalmed7100281)
Supplement: Supplementary file 1 [file tropicalmed-07-00281-s001.zip › tropicalmed-1892949-supplementary.pdf]

Supplementary Material

Table S1. Distribution of resistance pattern of *E. coli* to different antimicrobial agents according to the infection type.

Antibiotics	Wound infections	Ear infections	Burns infections	Chest infections	UTIs	Gastroenteritis	ICU	% Resistant
Cefpodoxime	22	2	4	6	5	6	5	83.3
Cefadroxil	25	3	5	7	6	6	6	96.7
Streptomycin	17	3	5	7	6	6	6	83.3
Aztreonam	12	1	3	4	6	6	5	61.7
Ceftriaxone	18	3	5	7	6	6	6	85
Cephalothin	26	3	5	7	6	6	6	98.3
Gentamycin	13	1	2	4	3	2	1	43.3
Amoxicillin/ Clavulanic	27	3	5	7	6	5	6	98.3
Ceftazidime	22	1	5	6	4	6	6	83.3
Imipenem	22	2	3	6	4	4	4	75
Meropenem	11	1	2	2	3	3	3	41.7
Cefoperazone	16	2	4	6	5	6	1	66.7
Doxycycline	23	3	4	6	5	6	6	88.3
Tetracycline	19	1	2	2	2	2	0	46.7
Ciprofloxacin	11	1	2	5	4	5	5	55
Amikacin	15	2	4	6	1	2	5	58.3
Nalidixic acid	14	2	4	7	6	5	6	73.3
Cefotaxime	25	3	4	7	6	6	6	95
Piperacillin	24	3	4	7	5	0	0	71.7
Cefepime	20	3	4	6	6	5	6	81.7
Ampicillin/sulbac tam	26	2	5	6	6	6	6	95
Norfloxacin	11	3	2	5	3	5	1	50
Tobramycin	14	2	4	1	0	0	1	36.7
Sulphamethoxazo le/trimethoprim	22	1	5	7	6	6	6	88.3
Nitrofurantoin	13	1	1	4	5	5	4	53.3
Chloranphenicol	12	2	4	5	3	2	1	48.3
Levofloxacin	13	2	3	1	0	1	0	33.3
Piperacillin/ Tazobactam	5	2	2	1	2	5	3	33.3
Ofloxacin	11	2	2	3	4	5	2	48.3
Azithromycin	16	2	1	5	4	0	1	50

Table S2. Distribution of bla_{IMP} genotype among 60 isolated *E. coli.*

E.coli	Source of sample	Resistance pattern of imipenem			*bla*$_{IMP}$
		Sensitive	Intermediate	Resistance	
1.	Wound	+	-	-	+
2.	Wound	-	-	+	+
3.	Wound	-	-	+	+
4.	Wound	-	-	+	+
5.	Wound	+	-	-	-
6.	Wound	-	-	+	+
7.	Wound	-	-	+	+
8.	Wound	-	+	-	-
9.	Wound	+	-	-	-
10.	Wound	-	-	+	+
11.	Wound	-	-	+	+
12.	Wound	-	-	+	+
13.	Wound	-	-	+	+
14.	Wound	-	-	+	+
15.	Wound	+	-	-	-
16.	Wound	-	-	+	+
17.	Wound	-	-	+	+
18.	Wound	-	-	+	+
19.	Wound	-	-	+	+
20.	Wound	-	-	+	+
21.	Wound	-	-	+	+
22.	Wound	-	-	+	+
23.	Wound	-	-	+	+
24.	Wound	-	-	+	+
25.	Wound	-	-	+	+
26.	Wound	-	-	+	+
27.	Wound	-	-	+	+
28.	Ear infection	+	-	-	-
29.	Ear infection	-	-	+	+
30.	Ear infection	-	-	+	+
31.	Burn	+	-	-	-
32.	Burn	-	-	+	+
33.	Burn	-	-	+	+
34.	Burn	+	-	-	-
35.	Burn	-	-	+	+
36.	Burn	-	-	+	+
37.	Burn	-	-	+	+
38.	Chest infection	-	-	+	+
39.	Chest infection	-	-	+	+
40.	Chest infection	+	-	-	-
41.	Urinary tract Infection	-	-	+	+
42.	Urinary tract Infection	-	-	+	+
43.	Urinary tract Infection	+	-	-	-
44.	Urinary tract Infection	-	-	+	+
45.	Urinary tract	-	-	+	+

	Infection				
46.	Urinary tract Infection	-	+	-	+
47.	Urinary tract Infection	-	-	+	+
48.	Urinary tract Infection	-	-	+	+
49.	Urinary tract Infection	-	-	+	+
50.	Urinary tract Infection	-	+	-	-
51.	Gastroenteritis	-	-	+	+
52.	Gastroenteritis	-	-	+	+
53.	Gastroenteritis	-	+	-	-
54.	Gastroenteritis	-	-	+	+
55.	Gastroenteritis	+	-	-	-
56.	Patients admitted to intensive care unit	-	-	+	+
57.	Patients admitted to intensive care unit	-	-	+	+
58.	Patients admitted to intensive care unit	-	+	-	+
59.	Patients admitted to intensive care unit	-	-	+	+
60.	Patients admitted to intensive care unit	-	-	+	+

Table S3. Distribution of *aac(6´)-Ib* genotype among 60 isolated *E.coli.*

E.coli	**Source of sample**	**Resistance pattern of amikacin**			***aac(6´)-Ib***
		**Sensitive**	**Intermediate**	**Resistance**	
1.	Wound	-	-	+	+
2.	Wound	+	-		+
3.	Wound	-	-	+	+
4.	Wound	+	-	-	-
5.	Wound	-	-	+	+
6.	Wound	+	-	-	+
7.	Wound	-	-	+	+
8.	Wound	-	-	+	+
9.	Wound	+	-	-	-
10.	Wound	-	-	+	+
11.	Wound	+	-	-	-
12.	Wound	-	-	+	+
13.	Wound	-	-	+	+
14.	Wound	+	-	-	-
15.	Wound	-	-	+	+
16.	Wound	+	-	-	+
17.	Wound	-	-	+	+
18.	Wound	+	-	-	-
19.	Wound	-	-	+	+
20.	Wound	+	-	-	-
21.	Wound	-	-	+	+
22.	Wound	+	-	-	-
23.	Wound	-	-	+	+

24.	Wound	+	-	-	+
25.	Wound	-	-	+	+
26.	Wound	+	-	-	-
27.	Wound	-	-	+	+
28.	Ear infection	-	-	+	+
29.	Ear infection	-	-	+	+
30.	Ear infection	+	-	-	-
31.	Burn	-	-	+	+
32.	Burn	-	-	+	+
33.	Burn	+	-	-	-
34.	Burn	-	-	+	+
35.	Burn	-	-	+	+
36.	Burn	-	-	+	+
37.	Burn	-	-	+	+
38.	Chest infection	+	-	-	-
39.	Chest infection	-	-	+	+
40.	Chest infection	-	-	+	+
41.	Urinary tract infection	-	-	+	+
42.	Urinary tract Infection	-	-	+	+
43.	Urinary tract Infection	-	+	-	+
44.	Urinary tract Infection	+	-	-	-
45.	Urinary tract Infection	-	+	-	-
46.	Urinary tract Infection	+	-	-	-
47.	Urinary tract Infection	-	-	+	+
48.	Urinary tract Infection	-	+	-	-
49.	Urinary tract Infection	-	+	-	-
50.	Urinary tract Infection	+	-	-	-
51.	Gastroenteritis	-	-	+	+
52.	Gastroenteritis	-	+	-	-
53.	Gastroenteritis	-	-	+	+
54.	Gastroenteritis	+	-	-	-
55.	Gastroenteritis	-	-	+	+
56.	Patients admitted to intensive care unit	-	-	+	+
57.	Patients admitted to intensive care unit	+	-	+	+
58.	Patients admitted to intensive care unit	-	+	-	-
59.	Patients admitted to intensive care unit	-	-	+	+
60.	Patients admitted to intensive care unit	-	-	+	+

Table S4. Time-killing curve for different groups infected with *E.coli* (wound no.3) resistant to both IMP & AMK .

		Control group (I)	Amikacin group (II)	Imipenem group (III)	Combination group (IV)	*p* value
0.5 mic concentration	**0 h**	8.20±0.02	8.20±0.02	8.20±0.02	8.20±0.02	*1*
	**2 h**	9.36±0.01	9.35±0.01	9.36±0.02	9.31±0.01	*<0.001**
	**4 h**	9.47±0.01	9.46±0.01	9.45±0.02	9.24±0.03	*<0.001**
	**8 h**	10.08±0.02	10.05±0.02	10.05±0.02	9.04±0.02	*<0.001**
	**12 h**	10.36±0.01	10.34±0.01	10.34±0	8.80±0.09	*<0.001**
	**24 h**	10.47±0.01	10.47±0	10.47±0	7.46±0.01	*<0.001**
1 mic concentration	**0 h**	8.20±0.02	8.20±0.02	8.20±0.02	8.20±0.02	*1*
	**2 h**	9.36±0.01	9.22±0.02	9.25±0.01	9.19±0.04	*<0.001**
	**4 h**	9.47±0.01	7.18±0.01	7.20±0.01	7.09±0.01	*<0.001**
	**8 h**	10.08±0.02	7.07±0.02	7.15±0.02	6.97±0.01	*<0.001**
	**12 h**	10.36±0.01	6.91±0.03	6.97±0.02	6.87±0.01	*<0.001**
	**24 h**	10.47±0.01	6.75±0.04	6.81±0.03	4.7±0.02	*<0.001**
2 mic concentration	**0 h**	8.20±0.02	8.20±0.02	8.20±0.02	8.20±0.02	*1*
	**2 h**	9.36±0.01	8.01±0.01	8.01±0.01	7.98±0.02	*<0.001**
	**4 h**	9.47±0.01	7.93±0.01	7.95±0.03	7.88±0.03	*<0.001**
	**8 h**	10.08±0.02	6.72±0.05	6.82±0.02	6.65±0.04	*<0.001**
	**12 h**	10.36±0.01	6.64±0.02	6.68±0.05	5.94±0.03	*<0.001**
	**24 h**	10.47±0.01	5.53±0.05	5.85±0.04	4 ±0.04	*<0.001**
4 mic concentration	**0 h**	8.20±0.02	8.20±0.02	8.20±0.02	8.20±0.02	*1*
	**2 h**	9.36±0.01	6.87±0.06	6.89±0.02	6.72±0.05	*<0.001**
	**4 h**	9.47±0.01	5.64±0.08	5.80±0.01	4.60±0.04	*<0.001**
	**8 h**	10.08±0.02	4.50±0.03	4.63±0.03	0±0	*<0.001**
	**12 h**	10.36±0.01	0±0	0±0	0±0	*<0.001**
	**24 h**	10.47±0.01	0±0	0±0	0±0	*<0.001**

* Significant p-value: <0.001.

Table S5. Time-kill studies for different groups infected with imipenem resistant *E. Coli* (W2).

	.	Control group (I)	Amikacin group (II)	Imipenem group (III)	Combination group (IV)	*p* value
0.5 mic concentration	**0 h**	8.20±0.02	8.20±0.02	8.20±0.02	8.20±0.02	*1*
	**2 h**	9.36±0.01	6.79±0.04	9.01±0.01	8.92±0.03	*<0.001**
	**4 h**	9.47±0.01	5.61±0.07	9.06±0.01	8.71±0.06	*<0.001**
	**8 h**	10.08±0.02	5.58±0.06	9.10±0.01	8.02±0.01	*<0.001**
	**12 h**	10.36±0.01	4.64±0.07	9.22±0	8.26±0.01	*<0.001**
	**24 h**	10.47±0.01	4.50±0.01	10.2±0.01	8.09±0.01	*<0.001**
1 mic concentration	**0 h**	8.20±0.02	8.20±0.02	8.20±0.02	8.20±0.02	*1*
	**2 h**	9.36±0.01	6.25±1.22	8.95±0.02	6.87±0.02	*<0.001**
	**4 h**	9.47±0.01	5.86±0.01	8.70±0.05	6.53±0.02	*<0.001**
	**8 h**	10.08±0.02	4.52±0.02	7.90±0.03	5.78±0.03	*<0.001**
	**12 h**	10.36±0.01	0±0	6.69±0.04	5.49±0.01	*<0.001**
	**24 h**	10.47±0.01	0±0	6±0.02	4±0.03	*<0.001**

* Significant p-value: <0.001.

Table S6. Time-kill studies for different groups infected with amikacin resistant *E. Coli* (W1).

		Control group (I)	Amikacin group (II)	Imipenem group (III)	Combination group (IV)	*p* value
0.5 mic concentration	**0 h**	8.20±0.02	8.20±0.02	8.20±0.02	8.20±0.02	*1*
	**2 h**	9.36±0.01	9.28±0.01	6.69±0.04	9.25±0.02	*<0.001**
	**4 h**	9.47±0.01	9.32±0.01	5.72±0.06	9.21±0	*<0.001**
	**8 h**	10.08±0.02	9.45±0.01	5.48±0.01	9.01±0.01	*<0.001**
	**12 h**	10.36±0.01	9.31±0.41	4.72±0.05	8.88±0.01	*<0.001**
	**24 h**	10.47±0.01	10.01±0.01	4.51±0.03	8.03±0.02	*<0.001**
1 mic concentration	**0 h**	8.20±0.02	8.20±0.02	8.20±0.02	8.20±0.02	*1*
	**2 h**	9.36±0.01	7.95±0.02	5.58±0.05	6.93±0.01	*<0.001**
	**4 h**	9.47±0.01	7.67±0.03	4.59±0.07	6.60±0.04	*<0.001**
	**8 h**	10.08±0.02	6.85±0.03	0±0	5.87±0.03	*<0.001**
	**12 h**	10.36±0.01	6.52±0.03	0±0	5.50±0.03	*<0.001**
	**24**	10.47±0.01	6±0.02	0±0	4±0.04	*<0.001**

* Significant p-value: <0.001.

In-vivo studies

Table S7. Average blood bacterial count of E. coli isolated from untreated animals and animals treated with imipenem, amikacin, and combination at different time.

Organism	Time	Untreated (I)	Imipenem (II)	Amikacin (III)	Combination (IV)	*p* value
E. coli	3h	9.41±0.01	9.34±0.01	9.28±0.01	9.11±0.02	<0.001*
	11h	10.29±0.02	9.05±0.02	8.84±0.05	8.13±0.03	<0.001*
	27h	10.36±0.01	8.71±0.06	8.25±0.01	6.0±0.52	<0.001*

p values between different times.

	3h vs 11h	3h vs 27h	11h vs 27h
**Untreated**	<0.001*	<0.001*	<0.001*
**Imipenem**	<0.001*	<0.001*	<0.001*
**Amikacin**	<0.001*	<0.001*	<0.001*
**Combination**	<0.001*	<0.001*	<0.001*

Table S8. Gene expression of resistant *E.coli* after treatment.

	16S rRNA	***bla-IMP***		***aac(6′)-Ib***	
E.coli **Isolate No.**	**CT**	**CT**	**Fold change**	**CT**	**Fold change**
**E1 (Control)**	20.57	22.38	-	23.04	-
**E2**	19.88	18.49	9.1896	19.73	6.1475
**E3**	20.31		Nd	19.31	11.0809
**E4**	20.16	18.19	13.7370		Nd
**E5**	19.54	19.53	3.5328	19.60	5.3147
**E6**	20.20	19.50	5.6962	21.23	2.7132

E1: Control.

E2: *E.coli* (w3) resistant for amikacin harboring aac(6′)-Ib and imipenem harboring bla_{IMP}

E3: *E.coli* (W1) resistant to amikacin harboring aac(6′)-Ib only.

E4: *E.coli* (W2) resistant to imipenem harboring bla_{IMP} only.

E5: *E.coli* isolate (w3) after treament with 0.25MIC of amikacin+0.5MIC of imipenem.

E6: *E.coli* isolate (w3) after treament with 0.25MIC of imipenem+0.5MIC of amikacin.
